# Urine biomarkers of renal renin–angiotensin system activity: Exploratory analysis in humans with and without obstructive sleep apnea

**DOI:** 10.14814/phy2.14376

**Published:** 2020-03-24

**Authors:** Patrick J. Hanly, Sofia Ahmed, Chris D. Fjell, George B. Handley, Darlene Sola, David Nicholl, Ann Zalucky

**Affiliations:** ^1^ Department of Medicine University of Calgary Calgary AB Canada; ^2^ Sleep Centre Foothills Medical Centre Calgary AB Canada; ^3^ Hotchkiss Brain Institute University of Calgary Calgary AB Canada; ^4^ Libin Cardiovascular Institute University of Calgary Calgary AB Canada; ^5^ Alberta Kidney Disease Network Canada; ^6^ True Bay Technologies Inc British Columbia Centre for Disease Control Public Health Laboratory Vancouver BC Canada; ^7^ Healthy Heart Sleep Company Calgary AB Canada

**Keywords:** CPAP, hypoxemia, kidney, renin–angiotensin system, sleep apnea, urine analytes

## Abstract

Obstructive sleep apnea (OSA) may contribute to kidney injury by activation of the renin–angiotensin system (RAS), which is reduced by continuous positive airway pressure (CPAP) therapy. A biomarker in the urine that reflects renal RAS activity could identify patients at risk of kidney injury and monitor their response to CPAP therapy. Nine patients with OSA and six matched control subjects without OSA were recruited. Renal RAS activity was measured by the renovasoconstrictor response to Angiotensin II challenge, a validated marker of RAS activity, and urine samples were collected in all subjects at baseline and repeated in those with OSA following treatment with CPAP. A broad range (1,310) of urine analytes was measured including 26 associated with the RAS signaling pathway. The OSA group was a similar age and weight as the control group (48.7 ± 10.4 vs. 47.7 ± 9.3 yrs; BMI 36.9 ± 7.2 vs. 34.7 ± 2.5 kg/m^2^) and had severe sleep apnea (ODI 51.1 ± 26.8 vs. 4.3 ± 2/hour) and nocturnal hypoxemia (mean SaO_2_ 87 ± 5.2 vs. 92.6 ± 1.1%). CPAP corrected OSA associated with a return of the renovasocontrictor response to Angiotensin II to control levels. Partial least squares (PLS) logistic regression analysis showed significant separation between pre‐ and post‐CPAP levels (*p* < .002) when all analytes were used, and a strong trend when only RAS‐associated analytes were used (*p* = .05). These findings support the concept that urine analytes may be used to identify OSA patients who are susceptible to kidney injury from OSA before renal function deteriorates and to monitor the impact of CPAP therapy on renal RAS activity.

## Graphical abstract

Obstructive sleep apnea (OSA) is common in patients with chronic kidney disease (CKD) and may contribute to kidney injury by activation of the renin–angiotensin system (RAS). In this study we report how CPAP reduced RAS activity in OSA patients associated with a significant separation in a broad range of urine analytes including those associated with the RAS signaling pathway. We propose that urine analytes may be used to identify OSA patients who are susceptible to kidney injury from nocturnal hypoxemia and to monitor the impact of CPAP therapy.

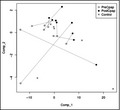

## 1 INTRODUCTION

The global prevalence of chronic kidney disease (CKD) is increasing and currently it occurs in more than 10% of adults (Editorial, 2013; Eckardt, Coresh, & Devuyst, 2013 ^)^ Chronic kidney disease increases the risk of cardiovascular morbidity and mortality (Go, Chertow, Fan, McCulloch, & Hsu, 2004) and progression of CKD to renal replacement therapy carries an enormous economic burden (Foley, Gilbertson, Murray, & Collins, 2011; Levey et al., 2007). Activation of the renin–angiotensin system (RAS) leads to kidney damage (Hostetter, 2003) by facilitating systemic hypertension (Foster et al., 2010), glomerular hyperfiltration, inflammation, and ultimately fibrosis within the kidney (Ruggenenti, Cravedi, & Remuzzi, 2012; Ruiz‐Ortega et al., 2006). Consequently, one of the main strategies to prevent progression of CKD is inhibition of the RAS with medications, such as angiotensin‐converting enzyme inhibitors (ACEIs) and angiotensin‐receptor blockers (ARBs). Some patients may benefit from higher doses of these medications, independent of BP effects (Miller et al., 2006), but some still progress to end‐stage kidney disease (ESKD) despite maximal therapy. This highlights the need both to identify RAS activity at the point of care and to seek additional therapeutic options for RAS inhibition.

Sleep apnea occurs in up to 40% of patients with CKD (Nicholl et al., 2012) and may contribute to the pathogenesis of kidney failure, partly through activation of the RAS. Nocturnal hypoxemia due to OSA has been associated with an accelerated loss of kidney function (Ahmed et al., 2011), and there is evidence from experimental animal (Fletcher, Orolinova, & Bader, 1985) and human (Foster et al., 2010) models that the systemic RAS is activated by intermittent hypoxemia. We have shown in patients with OSA, who had normal kidney function, that nocturnal hypoxemia increases RAS activity in the kidney (Zalucky et al., 2015) and that this is corrected by treatment with continuous positive airway pressure (CPAP) (Nicholl et al., 2014).

In a study designed to investigate the effect of CPAP therapy for OSA on the renal RAS (Nicholl et al., 2014), we performed an exploratory investigation of the change in urine biomarkers, including some associated with the RAS signaling pathway, following correction of OSA and nocturnal hypoxemia. Specifically, we compared urine analytes in men and women with OSA before and after treatment with CPAP in addition to a healthy, matched control group. The objective of this study was to determine if these biomarkers changed in a consistent manner following treatment of OSA with CPAP.

## 2 METHODS

### 2.1 Participants

Subjects with OSA were recruited from community patients referred for suspected OSA to the Foothills Medical Centre Sleep Centre and to respiratory homecare companies in Calgary, AB, Canada, between June 2011 and June 2014. Men and women, aged 18–70, with moderate‐to‐severe OSA and significant nocturnal hypoxemia (defined below) were eligible to participate in the study. Exclusion criteria included cardiovascular, cerebrovascular, and kidney disease, uncontrolled hypertension (BP > 140/90 despite maximal use of antihypertensive medications), diabetes mellitus, severe lung disease, current treatment for OSA, current smoking, pregnancy, use of nonsteroidal anti‐inflammatory medications, or exogenous sex hormones. A control group of obese subjects without OSA, confirmed by a home sleep apnea test (HSAT), was recruited from the community. All subjects underwent a medical history, physical examination, and laboratory screening. Twenty‐five subjects with OSA and 13 obese controls completed the study and the primary outcome of change in renal RAS in response to CPAP has been reported elsewhere (Nicholl et al., 2014; Zalucky et al., 2015).

Due to budgetary constraints, we could only analyze 24 urine samples. Consequently, we chose nine OSA patients in whom renal RAS activity fell after CPAP therapy and six control subjects who had renal RAS activity measured at the time of their HSAT. The study was approved by the Conjoint Health Research Ethics Board at the University of Calgary. All subjects provided written informed consent.

### 2.2 Determination of obstructive sleep apnea status

Both OSA patients and control subjects performed an unattended HSAT at home (Remmers Sleep Recorder (RSR) Model 4.2, Saga Tech Electronic, Calgary, AB, Canada). The monitor consists of an oximeter to record oxyhemoglobin saturation (SaO_2_) and heart rate variability, pressure transducer to record nasal airflow, microphone to record snoring, and a body position sensor. The oximeter provides the data for an automated scoring algorithm, which calculates the oxygen desaturation index (ODI) based on the number of episodes of oxygen desaturation > 4%/hr of monitoring. The Remmers Sleep Recorder has been validated by comparison to attended polysomnography (Issa et al., 1993; Vazquez et al., 2000). Sleep apnea was defined as ODI ≥ 15 and nocturnal hypoxemia was defined as SaO_2_ < 90% for ≥ 12% of the recording time, which has been used previously (Nieto et al., 2000). The raw data were reviewed by a sleep medicine physician (PJH) who confirmed that the estimated ODI was accurate and diagnostic of OSA.

### 2.3 Treatment of OSA with CPAP

After completing the first study day, control subjects were discharged and patients with OSA were treated with CPAP therapy. All subjects underwent an auto‐CPAP trial to determine their individual CPAP requirement. Initial auto‐CPAP settings were 16/6 cm H_2_O and were automatically titrated according to the CPAP unit titration algorithm to optimize therapy. If airflow limitation or nocturnal hypoxemia were not fully corrected, subjects were switched to fixed CPAP, which was estimated from the CPAP level at the 95th percentile. Adherence to CPAP therapy was monitored by electronic download from the unit each month. Once satisfactory CPAP adherence was achieved (defined as CPAP use for > 4 hrs/night on > 70% nights for 4 weeks) and correction of OSA and nocturnal hypoxemia was confirmed by a repeat HSAT while using CPAP, subjects with treated OSA underwent reassessment of renal RAS activity during a second study day, identical to the pre‐CPAP assessment.

### 2.4 Measurement of renal RAS activity

The study protocol for the assessment of renal RAS activity is well established and validated (Shoback et al., 1983). In brief, the renal plasma flow (RPF) response to AngII challenge is a surrogate marker of renal RAS activity, where a more blunted response represents upregulation of the renal RAS. Subjects were instructed to consume > 200mmol sodium per day for 3 days before the study day to ensure maximum RAS suppression. Subjects were studied in the supine position in a temperature‐controlled, quiet room after an 8‐hr fast. All subjects provided a second morning void spot urine for verification of diet compliance and determination of urinary sodium (Kawasaki, Itoh, Uezono, & Sasaki, 1993). Subjects on medications interfering with RAS activity discontinued their medications and changed to a calcium‐channel blocker (amlodipine) at doses to achieve adequate blood pressure control 2 weeks prior to the study day, as this agent is considered to have a neutral effect on the RAS (Sasaguri et al., 1997). The RAS‐inhibitor medication was restarted immediately after the study day.

At 8 a.m., an 18‐gauge peripheral venous cannula was inserted into each antecubital vein (1 for infusion, 1 for blood sampling). Each subject was given a loading dose 8mg/kg of paraaminohippurate (PAH; Merck, Canada) and 50 mg/kg of Inutest (Clinalfa, Austria), followed by constant infusions of PAH at 12 mg/min and Inutest at 30mg/min for 90 min to establish RPF and glomerular filtration rate (GFR), respectively. Filtration fraction (FF), a surrogate marker of glomerular pressure, was calculated as GFR/RPF. After a 90 min equilibration period, renal hemodynamics (RPF, GFR, and FF) and BP were measured at baseline and in response to a graded AngII infusion (3 ng kg^‐1^ min^−1^ × 30 min, 6 ng kg^−1^ min^−1^ x 30min) as an index of RAS activity (Nicholl et al., 2014). Blood samples were collected at baseline, and after each AngII infusion. Blood pressure was recorded every 15 min by an automatic recording device (Dinamap; Critikon). Subjects were studied in the supine position using a standard cuff placed on the right arm. The mean of two readings taken by the same Registered Nurse (DYS) was recorded.

### 2.5 Collection and analysis of urine samples

Urine samples were collected when subjects attended the research laboratory prior to their assessment of renal RAS. At approximately 8a.m., subjects urinated into a sterile cup. The sample was immediately placed in 4 degrees Celsius and was aliquoted 4 hr later into 2‐ml tubes and placed in a freezer set at a minus 70 degrees for later analysis of urine analytes (SOMAscan^®^ Boulder) using a highly sensitive multiplexed biomarker discovery platform which consists of 1,310 SOMAmer (Slow Off‐rate Modified Aptamer) reagents developed to bind human proteins (Gold et al., 2010). The 1,310 targets in the assay represent proteins known to be important in human diseases including cytokines (20%), growth factors (13%), receptors (21%), proteases (17%), protease inhibitors (5%), kinases (20%), structural proteins (1%), and hormones (3%). Of the target proteins 47% are secreted proteins, 28% are extracellular domains of membrane‐bound proteins, and 25% are intracellular proteins. SOMAmer reagents are made from single‐stranded DNA (ssDNA) that is chemically modified to mimic amino acid side chains. These modifications dramatically improve the binding of SOMAmer reagents to their target proteins (Rohloff et al., 2014). SOMAmer reagents are generated through a proprietary and patented process, called SELEX (Systematic Evolution of Ligands by Exponential Enrichment), that incorporates non‐natural nucleotide bases with amino acid–like side chains on the 5’ position of the uracil ring. This addition of amino acid–like side chains has been shown to significantly increase SOMAmer reagent physiochemical diversity, contributing to more stable intramolecular folding and to intermolecular interactions, leading to increased affinity and specificity for its protein target and slower SOMAmer‐target dissociation.

Creatinine levels were measured in urine samples to correct for the effect of urine concentration or dilution on the measured analyte level. Accordingly, analyte levels were normalized by dividing the measured value by the urine creatinine level.

## 3 STATISTICAL ANALYSIS

### 3.1 Intergroup comparisons

Differences in patient demographics, CPAP adherence, HSAT results, blood pressure, and renal hemodynamics between OSA patients pre‐ and post‐CPAP and control subjects were analyzed by Student's *t* test.

### 3.2 Identification of signal for analytes affected by CPAP

Due to the large number of analytes and small number of samples, variable reduction and logistic regression were performed using Partial Least Squares (PLS) and the R package, plsRglm (R version 3.4.4, plsRglm version 1.1.1) (Bertrand, Bastien, Meyer, & Maumy‐Bertrand, 2014), respectively. The Spearman rank correlation (in R using *cor.test* with *method='spearman'*) was used to evaluate the correlation between the change in RPF before and after CPAP and the change in analytes, including those associated with the RAS signaling pathway according to curated gene sets associated with RAS signal transduction (details below in section: Evaluating importance of RAS pathway) before and after CPAP; *p*‐values were corrected for multiple testing using False Discovery Rate (Benjamini‐Hochberg method) (in R using *p.adjust* with *method=’fdr’*). While there were differences in analyte values between groups, none were significant after adjusting for multiple testing and could not be distinguished from chance variation (minimum *p* = .00068 by Mann‐Whitney U test for IFNA2, but *p* = 1 after adjustment using fdr).

Analyte values were scaled to mean of 0 and standard deviation of 1. A PLS logistic regression model was built from pre‐ and post‐CPAP data (excluding controls) with the method, *plsRglm*, using pre‐/post‐CPAP indicators (0/1) as outcome variable (*data Y* parameter), scaled analyte values as predictor variables (*data X* parameter), model selector "pls‐glm‐logistic" (*modele* parameter), and two components (*nt* parameter). To assess the modeling method and estimate performance against data outside the dataset, leave‐one‐out cross‐validation was performed (*cv.plsRglm* method, with *K* = total number of Pre‐ and Post‐CPAP samples). To address difficulties of perfect separation in logistic regression, the *safeBinaryRegression* package( Konis, 2013) was used to test for the existence of the maximum likelihood estimate.

A biplot graph was plotted for each sample using analyte values projected onto the first two coordinates of the PLS model (the *tt* matrix of the model). The two components for control samples were calculated directly from scaled control analyte values multiplied by the *wwetoile* matrix in the PLS model. Leave‐one‐out cross‐validation (LOO CV) was performed to assess expected model performance, where separate models are calculated for the dataset with one data point removed (left‐out) and the left‐out sample value is then predicted. The contribution of each analyte to components is given by the loadings (*wwetoile* matrix), and as square root of sum of squared coefficients for the two components.

### 3.3 Permutation test for significance

As unique computational solutions were not always found (the maximum likelihood estimate did not exist), statistical significance was calculated using a permutation test as usual: group labels (“Pre‐CPAP” or “Post‐CPAP”) were randomly shuffled (i.e., sampled without replacement from the original set of labels under consideration). The permuted group labels remove any association between measured values and outcome, and thus represent empirical results expected by pure chance. Permutation and recalculation were performed 1,000 times to generate a large distribution of random results. By ranking results obtained from actual group labels to results gained from permuted group labels, empirical *p*‐values (**permutation test *p*‐values**) were obtained (e.g., if a result was better than 2% of results based on chance, the p‐value from permutation was 0.02). To assess permutation test *P*‐values, the accuracy of assigning sample labels (pre‐ and post‐CPAP) was used as the metric (e.g., if the accuracy of the actual model was better than 98% of the accuracy of models built on randomized outcome data, this would give a permutation *p*‐value of .02).

### 3.4 Evaluating importance of RAS pathway

To assess whether the analytes involved in the RAS pathway were particularly effective in distinguishing pre‐ and post‐CPAP samples, a plsRglm model was calculated using only those analytes associated with RAS signal transduction. Analytes were matched to genes sets annotated in MSigDB (Broad Institute, Molecular Signatures Database v6.2, http://software.broadinstitute.org/gsea/msigdb) for RAS signal transduction pathway itself, or as regulating the RAS pathway. For GO_RAS_PROTEIN_SIGNAL_TRANSDUCTION, 15 analytes (AIF1, BCL6, CDK2.CCNA2, CDK2.CCNA2, CFL1, DBNL, FGF2, IGF1, JUN, KPNB1, MAPK11, MAPKAPK2, MAPKAPK3, MAPKAPK5, and PLAT) matched of 143 genes, and for GO_REGULATION_OF_RAS_PROTEIN_SIGNAL_TRANSDUCTION, 14 analytes (APOA1, APOE, BCL6, CAMK2D, CDH2, CRK, CSF1, FGF10, IGF1, ITGA1.ITGB1, NTRK1, RAC1, TGFB2, and TIMP2) matched of 186 genes, for a total of 26 unique analytes of 305 unique genes. A permutation *p*‐value was calculated as before, using the LOO CV accuracy as the measure of performance for predicting Pre‐ and Post‐CPAP samples using only the 26 analytes.

## 4 RESULTS

### 4.1 Subject characteristics

Patient demographics, co‐morbidities, and medications are shown in Table 1. The OSA and control subjects were well matched for age, gender, race, and BMI. All subjects were nondiabetic, nonsmoking, with normal kidney function, and had BP < 140/90 mmHg. Four of the OSA patients had a history of hypertension and were taking cardiac medications. However, any patient who was taking an angiotensin‐converting enzyme inhibitor, angiotensin receptor blocker or beta‐blocker was changed to a calcium channel blocker (Amlodipine) so that they were free of RAS‐interfering medications at the time that RAS activity was assessed.

**Table 1:** Patient demographics, medications, and CPAP adherence

	OSA Group *N* = 9	Control Group *N* = 6	*p*‐value
Age, years	48.7 ± 10.4	47.7 ± 9.3	.852
Male:Female	6:3	3:3	
Race, %Caucasian	89%	100%	
BMI, kg/m^2^	36.9 ± 7.2	34.7 ± 2.5	.418
*Medications, % of group*			
ACEI^a^	44%	0%	
Beta Blocker^a^	22%	0%	
Ca Channel Blocker	11%	0%	
*CPAP adherence, 3 months*			
% of nights CPAP used	94.9 ± 8.7		
>4 hr/night, % time	82.4 ± 18.7		
Average CPAP use, hrs/night	6.4 ± 1.1		
AHI (CPAP), events/hr	1.8 ± 1.2		

Abbreviations: ACEI, Angiotensin‐converting enzyme inhibitor; AHI, Apnea–Hypopnea Index; BMI, body mass index kg/m^2^; CPAP, continuous positive airway pressure.

a Changed to a calcium channel blocker (Amlodipine) during RAS measurements (see text for details).

### 4.2 CPAP therapy

Patients with OSA received treatment with CPAP and their adherence with this therapy is shown in Table 1 based on download from their CPAP units for 3 months prior to RAS reassessment. By all conventional measures, these results reflect that patients were adherent with therapy. Furthermore, nocturnal monitoring with a HSAT was repeated on CPAP, which confirmed that OSA and nocturnal hypoxemia were corrected and indistinguishable from those indices in the control group (Table 2). Similarly, blood pressure fell to the values recorded in control subjects following treatment with CPAP (Table 2).

**Table 2:** Sleep study and blood pressure in OSA patients and control group

	OSA Pre CPAP	OSA on CPAP	Control Group	Pre‐ versus. CPAP	Pre‐ versus. Ctrls	CPAP versus. Ctrls
ODI, events/hr	51.1 ± 26.8	2.8 ± 1.9	4.3 ± 2.0	0.001	0.001	0.166
SaO_2_ < 90%, % TRT	52.2 ± 26.5	5.9 ± 10.7	3.4 ± 4.6	0.003	0.001	0.602
Mean SaO_2_, %	87.0 ± 5.2	92.7 ± 1.6	92.6 ± 1.1	0.050	0.012	0.896
SBP, mmHg	129.6 ± 11.0	118.7 ± 12.7	122.3 ± 7.8	0.010	0.185	0.548
DBP, mmHg	75.9 ± 9.2	72.4 ± 8.3	72.7 ± 9.5	0.101	0.526	0.949
MAP, mmHg	93.8 ± 8.8	87.9 ± 9.2	89.2 ± 8.6	0.017	0.335	0.788

Abbreviations: DBP, diastolic blood pressure; MAP, mean arterial blood pressure; ODI, oxygen desaturation index; SaO_2_, oxygen saturation; SBP, systolic blood pressure; TRT, total recording time

### 4.3 Renal hemodynamics and renal RAS activity

Glomerular filtration rate was normal and similar between groups (Table 3). Prior to CPAP therapy, RPF tended to be lower and FF tended to be higher in OSA subjects and returned to control levels following correction of OSA and nocturnal hypoxemia with CPAP. The change in RPF following angiotensin infusion reflects renal RAS activity. A smaller change in RPF following angiotensin infusion reflects greater renal RAS activity. OSA patients had greater renal RAS activity prior to CPAP therapy, but this returned to control levels following effective CPAP. We also measured serum aldosterone levels, which fell with CPAP therapy from 231 ± 189 to 117 ± 38 pmol/L.

**Table 3:** Baseline renal hemodynamics and renal plasma flow response to Angiotensin II in OSA patients and control group

	OSA Pre‐CPAP	OSA on CPAP	Control Group	Pre‐ versus. CPAP	Pre‐ versus. Ctrls	CPAP versus. Ctrls
GFR, ml/min	109.2 ± 18.0	111.5 ± 11.2	107.0 ± 18.8	0.652	0.823	0.569
RPF, ml/min	710.3 ± 163.3	808.1 ± 166.7	806.6 ± 258.6	0.037	0.390	0.989
FF, %	15.7 ± 2.6	14.3 ± 2.9	13.8 ± 2.8	0.2301	0.201	0.746
Delta RPF T30‐T0, ml/min	−125.6 ± 97.1	−200.1 ± 88.1	−205.4 ± 140.0	0.001	0.213	0.929
Delta RPF T30‐T0, % baseline	−16.4 ± 10.7	−23.9 ± 6.3	−23.9 ± 7.2	0.008	0.158	1.000

Abbreviations: GFR, glomerular filtration rate; RPF, renal plasma flow; FF, filtration fraction; Delta RPF T30‐T0 = change in RPF during 30 min Angiotensin II infusion.

### 4.4 PLS model

**Figure 1:**
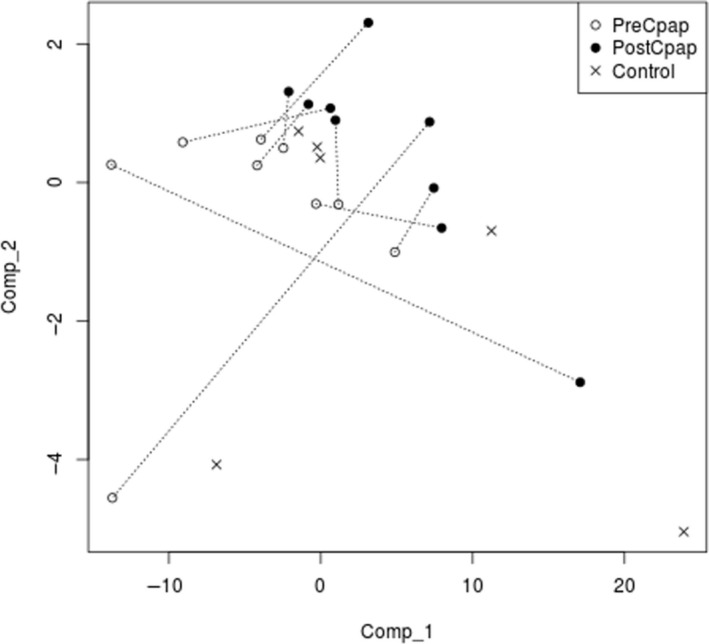
Biplot from PLSR logistic regression model. Comp_1 and Comp_2 are the top two components of the PLS model. Components are composed of a weighed combination of analyte values that best represent two sample types (Pre‐CPAP and Post‐CPAP). Each pair of dots represent a single patient's Pre‐ and Post‐CPAP data, transformed onto the two components, with dotted lines joining Pre‐ and Post‐CPAP samples for the same patient. Control patient samples are represented as x symbols

PLS Model: Partial Least Squares (PLS) logistic regression was used to model the difference between pre‐ and post‐CPAP. Two components were used to reduce the number of variables from the total number of analytes to two components, which are the axes of Figure 1. This figure shows the coordinates of each patient as two points, for pre‐ and post‐CPAP, based on the transformation of the analyte values into the dimensions of the PLS components; dotted lines connect the two points for each patient. With the pattern of post‐CPAP values being to the right and up from the pre‐CPAP values for each patient, interpretation of this difference in the context of analytes is not possible due to the complexity of the PLS model. However, the importance of each analyte in the overall model is reflected in the *loading* values of the model for each component (this reflects the contribution of the analyte to the components, outlined in Table S1: analyte CD207 contributed 20% to the overall loadings; an additional seven analytes contributed more than 1% [GCG, NTF4, PLK1, EIF4EBP2, MEPE, FGF2, and ADAM12]).

There is a risk of models performing well simply due to the small number of samples (patients) and large number of variables (analytes), regardless of how the model reflects reality. As can be seen in Figure 1, the points representing each patient, pre‐ and post‐CPAP, are clearly separated, and thus there is no unique optimal line (in the PLS component space) that divides them. For this reason, a unique set of best parameter estimates (maximum likelihood estimates) cannot be determined and confidence in the estimates cannot be reported directly. Instead, we assessed the confidence in the PLS model in two ways. First, using leave‐one‐out cross‐validation (LOO CV): one sample was omitted, and a model built on the remaining samples with the type of the left‐out sample predicted each time. Accuracy of the LOO CV models were 89%, confirming that PLS modeling was not overfitting the data. Secondly, we used a permutation test for significance to compare prediction of sample type (the pre‐ or postlabel) to what is achieved by random chance: Samples were randomly assigned to pre‐ and post‐CPAP types, in 1,000 trials, and each time the models were rebuilt and used to predict the sample types. As the sample types were random, the performance of these 1,000 trials represent what would be expected by a model where no actual relationship exists between analytes and pre‐ and post‐CPAP type. We found that the actual performance was better than 98% of the random trials, representing a permutation *p* = .02.

When only 26 analytes were used (those that are associated with the RAS signaling pathway), the accuracy was reduced to 89% (*p* = .05 by permutation test, and LOO CV accuracy of 0.72). While changes in individual analytes before and after CPAP were significantly correlated with the change in RPF following infusion of Angiotensin II, these may have been simply due to chance (the lowest *p* value was .007 for TIE1 and IL6; however, none were significant after adjustment for multiple testing with all *p* > .82).

## 5 DISCUSSION

In this study of human subjects with OSA, we have shown that a broad range of urine analytes change significantly when nocturnal hypoxemia due to OSA is corrected by CPAP therapy. Furthermore, there is a strong trend for a similar change in a smaller number of urine analytes involved in the RAS pathway following CPAP, which may reflect the associated downregulation of renal RAS in these patients. These findings support the concept that urine analytes may be used to identify patients with OSA who are susceptible to kidney injury from nocturnal hypoxemia before renal function deteriorates and, furthermore, to monitor the impact of CPAP therapy on the kidney.

Our study was limited by a small sample size, which impacts our ability to provide conclusive results. Furthermore, the small sample size and very large number of measured analytes preclude our ability to identify a specific analyte as a single biomarker of kidney injury; while several analytes had significant correlations between pre‐ and post‐CPAP values and the change in RPF before and after CPAP, none approached significance after adjusting for multiple testing. Nevertheless, the combination of our statistical analysis and well‐defined patient cohort facilitated our efforts to find a meaningful result. Specifically, our patients had severe OSA and nocturnal hypoxemia, which was corrected by CPAP. This effect was maintained for several months as reflected by the excellent adherence with CPAP therapy. Furthermore, renal RAS was upregulated in OSA patients and returned to normal (control) levels following CPAP therapy. Our OSA patients did not have active comorbid disease and had normal renal function, which minimized the potential for factors other than the changes in nocturnal hypoxemia to confound our results.

Another potential limitation was the control group we chose. Baseline measurements of renal RAS activity, hemodynamics, and urine analytes in an age‐ and weight‐matched group who did not have OSA or nocturnal hypoxemia provided valuable targets for the efficacy of CPAP therapy. However, follow‐up measurements at a time matched to those in the CPAP group would have strengthened our analysis. It would also be interesting to investigate the change in urine analytes in OSA patients in whom RAS activity did not fall with CPAP therapy. These options were not feasible in this study but could be considered in future research.

Single analyte predictive models are suggestive but none are statistically significant after correcting *p*‐values for multiple testing. However, the PLSR model shows a highly statistically significant difference between pre‐ and post‐CPAP analyte levels using all analytes (*p* < .002). Considering only RAS‐associated analytes, the statistical significance is borderline (*p* = .05) for the PLSR model.

Further studies are required to evaluate the role of urine analytes as potential biomarkers of kidney injury due to nocturnal hypoxemia. We favor the investigation of a smaller number of analytes that are reflective of pathways involved in the pathogenesis of kidney injury in a larger cohort of patients with OSA. The ultimate goal is to have the ability to identify OSA patients who are at risk of kidney injury from nocturnal hypoxemia in a noninvasive and inexpensive way, before renal function deteriorates, which would facilitate the development of a personalized approach to this clinical challenge.

## CONFLICT OF INTEREST

Dr Hanly has received financial support and equipment (CPAP units) from Philips Respironics for clinical research.
